# Ethnicity, Race and Skin Color: Challenges and Opportunities for Atopic Dermatitis Clinical Trials

**DOI:** 10.3390/jcm12113805

**Published:** 2023-06-01

**Authors:** Robert Bissonnette, Jasmina Jankicevic, Etienne Saint-Cyr Proulx, Catherine Maari

**Affiliations:** Innovaderm Research Inc., 3530 St-Laurent St, Suite 300, Montreal, QC H2X 2V1, Canada

**Keywords:** atopic dermatitis, eczema, ethnicity, race, skin of color, clinical trial

## Abstract

The number of clinical trials conducted in patients with atopic dermatitis is increasing steadily. These trials are conducted in several countries across all continents and include patients of different ethnicity, race and skin color. This diversity is desired, but it also brings challenges, including the diagnosis and evaluation of disease severity in patients with different skin colors; the influence of ethnicity on the perception of quality of life and patient reported outcomes; the inclusion of ethnicities that are only present in one country or that live far from clinical research sites; and the reporting of drug safety information. There is a need to better train physicians on the evaluation of atopic dermatitis in patients with different skin colors and a need to improve the systematic reporting of ethnicity, race and skin color in clinical trial publications.

## 1. Reporting of Ethnicity, Race and Skin of Color in Clinical Trials

Access to detailed information on baseline demographics is important to understand the outcome of clinical trials. Ethnicity, race and skin color are three descriptors often reported in AD clinical trials. Unfortunately, there is no consensus on the definition of these descriptors and how they should be used to characterize patient populations in clinical trials [1]. According to the Merriam Webster dictionary, race refers to groups that humans are often divided into, based on physical traits regarded as common among people of shared ancestry [2]. Race is dependent on genetic background and studies have shown that there are differences between races in terms of the relative importance of inflammatory pathways in AD [3]. The use of the word race to report findings in medical research has recently been criticized and ancestry has been proposed as a replacement [1]. Ethnicity is defined as affiliation to large groups of people classed according to common racial, national, tribal, religious, linguistic, or cultural origin [4]. People from the same ethnicity often are from the same race but this is not always the case. Ethnicity can have an important role on how patients perceive their skin disease and how it impacts their quality of life. Skin color can by defined by scales using visual examination and/or questionnaires, such as the Fitzpatrick’s phototype scale, or by measuring skin pigmentation with a chromameter. People with the same skin color can be of different races or different ethnicities. Demographic information about race, ethnicity and skin color is important to understand the results of clinical studies conducted in patients with atopic dermatitis. Unfortunately, race, ethnicity and skin color are rarely all included in published demographic tables from AD clinical studies and when they are, they are not reported in a standardized manner [5]. A comprehensive review of AD clinical trials published between 2014 and 2019 found that race and ethnicity were both reported in only 15.8% of publications [6]. The American Medical Association has recently published guidelines on how to report race and ethnicity in medical journals [7]. Similar guidelines on how to define and report race, ethnicity and skin color in clinical research protocols would be helpful.

Clinical trials must be conducted and reported in patients from different races, ethnicities and skin colors as these factors could have an effect on the safety and efficacy of drugs. This is especially important in atopic dermatitis, as differences in inflammatory pathways, disease severity, persistence and lesion morphology have been reported in patients from different racial backgrounds [3]. From a regulatory perspective, certain countries will require drugs to be tested in their main patient population before being approved. The regulatory authorities may require that a study is conducted in the country of their jurisdiction with subjects representative of that country’s patient population or may require a minimum of representative subjects from their country to be included in the pivotal trials. It is important for sponsors to understand the requirements of all countries targeted for regulatory submission and incorporate those requirements into their clinical development strategies and plans, especially for phase 3 programs.

## 2. Diagnostic Challenges

Most AD clinical studies conducted in North America and Europe use either the Hanifin and Rajka or the Eichenfield AAD criteria to establish a diagnosis of atopic dermatitis [8,9]. These criteria have not been studied in all ethnic and racial backgrounds and could therefore be less sensitive or specific in some populations. For example, the Hanifin–Rajka criteria have been shown not to perform optimally for the diagnosis of AD in certain non-white races [10,11]. The presence flexural lichenification and linearity in adults is one of the main factors considered in the Hanifin and Rajka diagnostic criteria. However, it has been reported that African American patients often have extensor, as opposed to flexor involvement [12]. African American patients have also been shown to have more prominent lichenification and a higher prevalence of prurigo nodularis [3,13]. Asian patients have also been reported to have more lichenification and sometimes present a psoriasiform morphology [12,14]. Interestingly, the use of Hanifin and Rajka criteria for diagnosing AD in Chinese patients has been criticized as being too stringent and other criteria have been developed [10,15]. However, Asian patients who live outside of Asia and African Americans are usually included in studies using the Hanifin and Rajka or the Eichenfield criteria [16,17,18].

## 3. Challenges in Measuring Treatment Efficacy

The main clinician-reported outcome measures used in AD research are EASI, SCORAD, investigator global assessments (IGAs), pruritus numerical rating scale and body surface area involved with atopic dermatitis. The Harmonization Outcome Measure for Eczema (HOME) group recommends the use of a combination of patient and clinician reported core outcome set in AD studies such as EASI, pruritus NRS, Objectif SCORAD, POEM, ADCT and RECAP [19]. Some of these outcome measures were validated in a diverse population including patients of different races, skin colors and ethnic backgrounds. However, rarely were validation studies conducted only in patients of a specific ethnicity, race or skin color, nor were these outcome measures compared between patients of different ethnicities, races or skin colors. Patient outcome measures, such as POEM, DLQI, ADCT and RECAP, may perform differently in patients of different ethnic backgrounds. Only a few studies have tried to address this question. Zhao et al. compared the inter and intra-rater reliability of EASI, SCORAD and IGA in patients with light skin and skin of color (defined by measuring melanin index by a chromameter) [20]. A total of five raters were given a 30-min training session and 25 patients were evaluated on two different days, including 14 patients with skin of color. All outcome measures were associated with excellent to good inter and intra-rater reliability. However, only EASI showed excellent inter-rater reliability. All assessors who participated in this study were experienced evaluators who regularly see AD patients. This is not always the case in clinical trials, where less experienced investigators, including non dermatologists or dermatologists who have a limited AD practice, are often the ones conducting efficacy evaluations. Sometimes, experienced evaluators practice in an area where they very rarely see patients with different races or skin color. The reliability of efficacy outcome measures should be studied with physicians who have more limited experience evaluating AD patients or less experience with the evaluation of patients of different races, ethnicities or skin colors. Interestingly, a study previously performed by the same group on photographs showed poor inter-rater reliability for highly pigmented patients [21].

Erythema is usually the first sign mentioned in the description section of the grades of various IGA scales. Additionally, it is the first sign mentioned in the list of signs from the original EASI and SCORAD publications [19,20]. Erythema is the most striking feature of AD in patients with phototype 1 to 3. Inflammation in lighter phototypes translates into redness. In darker skin phototypes, erythema is more difficult to evaluate and inflamed skin can appear gray or even purple (violaceous) [12,13]. When using clinical outcome measures in trials, investigators sometimes increase erythema by one level for patients with heavily pigmented skin [22]. However, this is not systematically done or suggested in clinical trials and the effect of this increase on the EASI scale performance has not been well studied. The differences in the clinical features of AD lesions between lighter skin and darker skin patients are summarized in Table 1.

The visual appearance of xerosis and dyschromia are other difficulties associated with the evaluation of patients with darker phototypes. Xerosis is not included in the IGA and EASI evaluations and ichthyosis is not included in IGA, EASI and SCORAD. The presence of xerosis and ichthyosis will reflect visible light and, therefore, the skin will appear whiter. White on dark is striking. Besides hyperpigmentation, such whiter appearance can be the most visible abnormality on the dark skin. It is important for evaluators not to include the effect of xerosis and ichthyosis when they perform EASI and IGA evaluations. This may be more difficult in patients with more heavily pigmented skin.

Excoriations may be more difficult to see in skin of color patients, whereas lichenification is usually more obvious [13]. Dyschromia is one of the predominant features of atopic dermatitis in patients with more heavily pigmented skin. Hypopigmentation and hyperpigmentation are not evaluated with the most frequently used AD clinician reported outcome measures. It is not rare to see patients with dark phototype who want to participate in a clinical trial and have only dyschromia with no evidence of active AD. This is problematic for several reasons. First, it is important for the clinician to have a close look at the skin of darker skin patients when evaluating AD in patients with significant dyschromia. Palpation is often very helpful to differentiate between patients with dyschromia without active AD and patients with active AD (distinction between flat skin—macules and raised skin—papules or plaques). Another important point is the paucity of AD studies that focus on improving dyschromia in patients with dark skin. This can be a cause of significant concern in darker-skinned patients and the prevention or treatment of hypopigmentation and hyperpigmentation is very rarely measured in clinical trials. Additionally, climate has an influence on skin lesions, as patients living in very hot and humid environments will have less xerosis but may have more red milliary [23]. The effect of climate on AD outcome measures used in clinical trials has not been well studied.

A few outcome measures have been adapted for patients with darker skin. This is the case of the Patient Oriented-SCORAD (PO-SCORAD), which has been adapted for patients with darker skin. This was done because the original PO-SCORAD had shown important test–retest differences between white and non-white patients [24]. A variant of EASI where erythema was replaced by a grey scale has been studied in patients with darker skin. Inter and intra-rater reliability has been shown to be good but was not very different from EASI performed with erythema evaluation [20].

A study looking at differential item functioning among patients of different races and ethnicities for various patients reporting outcome has shown differences in one or more items of several Patient Reported Outcomes (PROs) used regularly in AD research including DLQI, POEM and Itch NRS [5]. This is not surprising, as differences in culture could have a significant impact on disease perception. Genetic factors could also be involved, as shown by the differences in the molecular characteristics and intensity of pruritus in African American patients [25]. For DLQI, the most widely used PRO in dermatology, this was especially important for items number 2 (Over the last week, how embarrassed or self conscious have you been because of your skin?) and 10 (Over the last week, how much of a problem has the treatment for your skin been, for example by making your home messy, or by taking up time?).

## 4. Challenges in Evaluating Treatment Safety

There is a lack of studies on the effects of race and ethnicity on safety data generated from AD clinical trials. Some groups may be more or less inclined to report adverse events. In addition, safety data is rarely reported according to race or ethnicity in AD studies. For example, the risk of adverse events to anticoagulants, drugs used for diabetes and opioids, has been reported to be higher in Asian, Black and Caucasian patients, respectively [26]. The presence of the HLA-B*1502 allele has been shown to be associated with Stevens Johnson/toxic epidermal necrolysis in some Asian populations and screening has been recommended for patients at risk of carrying this allele [27]. There is a need to report safety data according to race and ethnicity.

## 5. Challenges of Generating Information from Patient Populations where the Drug Is Intended to Be Used

Clinical research sites in international studies conducted for the approval of novel AD treatments tend to be located mostly in North America, Europe and certain countries in the Asia Pacific region (e.g., China, Japan, Australia and South Korea). Africa, Latin America and the Middle East region usually have fewer sites and often no sites in international AD studies. Therefore, the data that are generated from international AD studies do not cover all ethnicities. Many countries have several major ethnic groups, with some representing a relatively small portion of the patient population. Even when many clinical research sites from such countries are included in clinical studies, it does not guarantee that the patient population recruited will include various ethnicities.

Willingness to participate in clinical trials also varies according to race and ethnicity. Asians living in North America have been shown to be less likely than African-Americans, Caucasians and Hispanics/Latinos to participate in clinical research [28]. A survey of clinical trials in AD published between 2014 and 2019, where at least one center was located in the US, showed that the proportion of White, Black, Asian and Hispanic patients was 62.4%, 25.3%, 7.4% and 16.3%, whereas the racial and ethnic distribution in the US at that time for these groups was 72.5%, 12.7%, 5.5% and 18% [6]. The proportion of White and Black patients in phase I and II trials was lower and higher, respectively, than in phase III trials. This suggests that representation of the main races and ethnicities in AD trials conducted in adults in the US may only sometimes be in line with the general US population. However, another study of AD trials conducted in children showed that the proportion of White, Black, Asian and Hispanic patients was 60.3%, 10.1%, 21.1% and 18.3% [29]. This suggests that Black patients may be under-represented in pediatric AD trials. In addition, challenges arise when patients of certain races and ethnicities live far from larger cities. Clinical research sites usually tend to be more frequently located in and around larger cities. This creates challenges for ethnic populations that do not live in larger cities. For example, there is a high burden of atopic dermatitis in Canadian indigenous people [30]. However, they tend to be underrepresented in clinical trials, as indigenous people often live hundreds if not thousands of kilometers from the nearest clinical research site.

Political instability and war are other factors limiting the ability to study new treatment in patients with more various ethnic backgrounds. For example, there were many active research sites in Ukraine and Russia that became unavailable after the Russo–Ukrainian war started in March 2022. Consequently, it will be difficult to generate efficacy and safety data for some of the new investigational products in the Ukrainian and Russian populations. Sponsors are usually hesitant to open research sites in unstable countries as they may not be able to complete their study or access the data, or investigators may not be able to ensure the safety of enrolled patients as shipments of blood or other samples to central labs and shipments of experimental drugs to research sites may be difficult.

Finally, when comparing safety and efficacy data from studies conducted in different countries, it is important to distinguish between differences related to race/ethnicity and differences in how clinical trials are conducted in these different countries. For example, the use of rescue medication has a dramatic influence on efficacy data in AD clinical studies. Two otherwise identical protocols with different strategies for rescue therapy or different estimates on how to treat intercurrent events, such as the use of rescue medication, could give rise to very different results. Differences between countries regarding the severity of the presence and extent of some clinical manifestations of AD could influence study results. The challenge of potential global differences in the conduct of clinical studies is best overcome by preventing such challenges through globally aligned implementation of best practices in clinical research of atopic dermatitis.

## 6. Conclusions and Recommendations

Race, ethnicity and skin color are rarely all reported in AD clinical trials. For a multifactorial and heterogenous disease such as AD, the lack of reporting on all three determinants limits the ability to deeply analyze clinical data and potentially discover what therapeutic options may be especially beneficial, or not, for certain subpopulations. An effort should be made to systematically report race, ethnicity and skin color used in all AD clinical trials. The recent American Medical Association guidelines on reporting race and ethnicity in medical journals offer an opportunity to standardize how safety and efficacy information is made available for these groups of patients.

## Figures and Tables

**Table 1:** AD charateristics in lighter and darker skin patients.

AD in Lighter Skin Patients	AD in Darker Skin Patients
Mostly flexural involvement	Extensor involvement often present
Psoriasiform morphology rarely present	Psoriasiform morphology often present (Asian patients)
Inflamed skin appears red	Inflammation often translates into greyish or violaceous (purple) color
Less lichenification	More lichenification
Excoriations are easily visible	Excoriations are often less visible
Xerosis is often less visible	Xerosis is usually more visible
Dyschromia is usually less present	Dyschromia if often the predominant feature
Prurigo nodularis is less frequent	Prurigo nodularis is more often present

## Data Availability

Not applicable.
